# Mesenchymal stem cells (MSC) delays the occurrence of graft-versus-host disease(GVHD) in the inhibition of hematopoietic stem cells in major histocompatibility complex semi-consistent mice by regulating the expression of IFN-γ/IL-6

**DOI:** 10.1080/21655979.2021.1955549

**Published:** 2021-07-24

**Authors:** Ying Wang

**Affiliations:** Department of Hematology, the Seventh Affiliated Hopital, Sun Yat-sen University, Shenzhen, China

**Keywords:** Interferon γ, mesenchymal stem cells, graft-versus-host hisease, hematopoietic stem cells

## Abstract

In recent years, because of its low immunogenicity and immunosuppression, mesenchymal stem cells (MSCs) have become a potential cell therapy for Graft-versus-host disease (GVHD). However, many experiments now focus on the interference of MSCs on T-cell proliferation in vitro and the prevention of GVHD in vivo. However, whether MSCs can effectively treat GVHD, the timing and conditions of treatment are not systematically studied. In order to clarify the therapeutic effect of MSC on GVHD, In this paper, mice were selected to build a model for study, and group control method was used. Experimental research proved that four mice died after transplantation with allogeneic hematopoietic stem cells treated by IFN- γ, and their white blood cell number remained basically unchanged, and their weight changed slightly. In addition, three groups of mice after allogeneic hematopoietic stem cell transplantation were used the incidence of GVHD was X^2^ = 20.6, indicating that the incidence of GVHD was significantly reduced and the survival rate of mice was significantly increased.

## GRAPHICAL ABSTRACT

**Figure:**
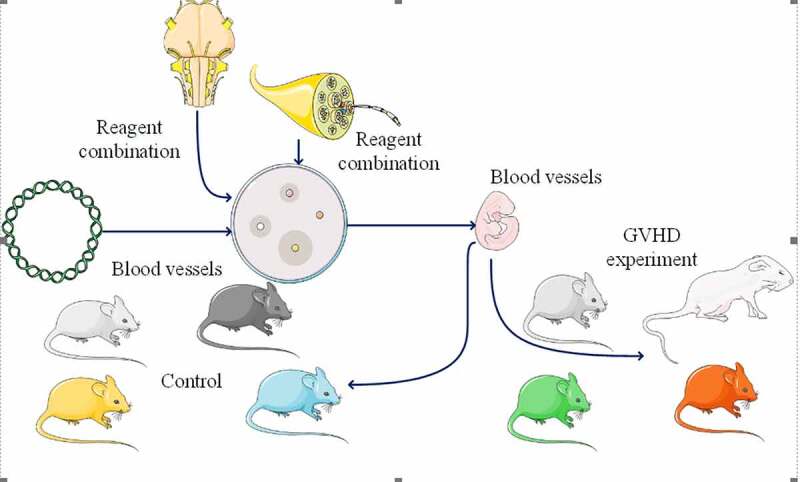

## Introduction

With the improvement of medical level, hematopoietic stem cell transplantation has been widely used in many hematological diseases, such as leukemia, lymphoma and simple anemia. However, complications such as graft-versus-host disease, delayed immunosuppression and recurrence of primary malignant tumors have become obstacles to long-term survival of patients after transplantation, which may lead to 30% – 40% of transplantation-related deaths. The success of allogeneic hematopoietic stem cell transplantation includes the reconstruction of hematopoietic function and immune function. The reconstruction of immune system enables patients to obtain the results of GVL. However, GVHD may occur when the donor cells invade multiple tissues and organs of the host and the body, and moderate or more GVHD will seriously affect the quality of life of patients. Therefore, it is of great clinical significance to study how to reduce the incidence and severity of GVHD, especially as an incomplete donor of stem cell receptor, and to improve the survival rate and quality of life after transplantation.

In today’s medical conditions, bone marrow mesenchymal stem cells (MSCs) are important research targets. They can secrete a variety of hematopoietic growth factors, reconstruct the blood microenvironment, and easily infect and express foreign genes. They are ideal target cells for tissue engineering, cell and gene therapy. Hülsdünker established GVHD model to study MSc [1,2]. He took the mice with different treatments as experimental materials, then irradiated them with radiation, then infused the mouse bone marrow mesenchymal stem cells cultured at the same time with IFN – γ into the mice after transplantation, and finally transplanted the mice with spleen cells, and observed the situation [3,4]. It was found that in the hematopoietic stem cell transplantation group, the mesenchymal stem cells could be differentiated into the middle layer cells of bone marrow, and even into cells similar to neurodegenerative cells and intradermal hepatocytes [5,6]. In addition, the differentiation of the damaged tissue, when generating stem cell colonization signal, and local microenvironment stimulation MSC, differentiation into functional specific tissue cells [7,8]. However, the number of experimental samples of this research method is too small, and the accuracy is difficult to convince the public.

At present, there are many studies on GVHD, for example, Yunjie found that the simultaneous infusion of BM-MSC into mice can significantly reduce GVHD [9,10]. In order to confirm his conjecture, he infused twenty unrelated HSCT patients with frozen bone marrow MSCs from unrelated third-party donors. Later, he found that the incidence of GVHD decreased, the overall survival rate of one year increased and the recurrence rate did not increase [11,12]. At the same time, the study also found that the treatment of umbilical cord MSc is very useful for the treatment of severe GVHD with glucocorticoid resistance, and can maintain a low recurrence rate of malignant hematopathy [13,14]. In addition, he also found that MSC can express Ido protein in vivo, proving that IDO activity changes under the induction of IFN – γ, so as to play a role in inhibiting T cell proliferation [15,16]. However, there are uncontrollable factors in this kind of experiment and the data are not reliable.

This article studies the inhibitory effect of MSC on GVHD in mice. In this experiment, we selected mice to establish a research model, isolated, purified and amplified the mouse GVHD model from the immunomagnetic bead separation of mouse bone marrow mesenchymal stem cells, and treated the mesenchymal stem cells with IFN-γ to infuse into mice, and further observed its effect on the inhibition of GVHD in mice.

## Materials and methods

### Bone marrow mesenchymal stem cells

Bone marrow mesenchymal stem cell (MSc) is a kind of multi-functional, spontaneous and multidirectional stem cell tissue. It can not only adjust the hematopoietic environment in vivo, but also promote the growth of hematopoietic stem cells. It has a broad application prospect in promoting blood reconstruction, tissue repair and gene therapy after bone marrow transplantation [17,18]. MSC can also inhibit the proliferation and secretion of T-lymphocyte factor and B-lymphocyte, and may reduce the incidence of graft-versus-host disease in clinic [19,20]. Therefore, as a promising alternative source of stem cell therapy, BMSCs have unlimited potential and broad clinical prospects, and their immunological characteristics are the decisive factors for the success of cell therapy [21].

### IFN-γ and IL-6

Interferon – γ (IFN – γ) and interleukin-6 (IL-6) are important inflammatory factors. They are the main pro-inflammatory factors secreted by activated T lymphocytes and NK cells. Mesenchymal stem cells can significantly reduce IFN – γ secretion of inflammatory cells [22,23]. In addition, IFN – γ can not only activate the immunoregulatory activity of mesenchymal stem cells, but also affect their proliferation and differentiation. Clinically, IFN – γ/IL-6 is often used for anti-virus, anti-tumor and immune response regulation [24,25]. Therefore, it is very important to understand the effect of IFN – γ

/ IL-6 on mesenchymal stem cells, to clarify the immune regulation mechanism of mesenchymal stem cells and its clinical application [26,27].

### Graft-versus-host disease

Graft-versus-host disease (GVHD) is a serious immunopathological disease, which is caused by the dysfunction of donor and recipient antigens after transplantation. Its occurrence is affected by many factors, mainly related to the incompatibility of human HLA. In the pathogenesis of GVHD, pretreatment before transplantation causes damage to the host tissue, which leads to the activation of ‘inflammatory storm factor’ and donor T cells, while the release of inflammatory substances leads to a large number of immune cells, such as NC cells, giant food cells, etc. donor T cells can also be differentiated into T cells with different functions due to different antigen stimulation, such as CD4 ^+^ T Cells, CD8 ^+^ T cells and regulatory T cells. The interaction among host cells, donor cells and antigen producing cells is a new immune balance network, which is often a positive immune response. If the number or function of one terminal cell is abnormal, it may lead to the imbalance of the whole immune balance network, thus reducing or worsening the whole. CD4 ^+^ T cells are the center of the immune system, the regulator and end point of the immune response. Therefore, blocking the immune response from the perspective of T cells may reduce the occurrence of GVHD to a certain extent, which is the main way to prevent graft-versus-host disease after transplantation.

### Experimental setup

In this study, 40 healthy BALB/c mice, aged 6–8 weeks and weighing 18–22 g, were selected as receptors. Model preparation and treatment plan: mice were irradiated by linear X-ray accelerator, the total dose was 8.5 gy, the dose rate was 0.5 gy/rain, and the radiation source was 50 cm away from animals. After radiation, they were raised aseptically in an air flow chamber. The bone marrow of femur and tibia was taken aseptically to make monocyte bone marrow cell suspension. The cell number was set to 1x10s/ml with rpml640 culture medium. At the same time, the spleen of mice was taken to prepare the spleen cell suspension and the cell number culture medium RPMI1640 was adjusted to 2x108/ml. 40 hours after irradiation, BALB/C of 40 rats was injected with 0,1 ml of rat bone marrow cell mixture and spleen (including 1h107 bone marrow cells and 2h106 spleen cells) at the same time Vein queue, randomly divided into four groups, the experiment. Five non irradiated rats were selected as the normal group, five irradiated mice as the negative control group. One ml of normal serum was taken and injected intravenously 4 hours after irradiation, and 40 mice were injected with 5 ml of bone marrow and spleen cells via tail vein 4 hours after irradiation. BM + PC + MSc group: 10 mice were injected with bone marrow cells and spleen cells, and then injected through tail vein every 4 hours, taking 0.5 ml of p6msc cells isolated from Macs; BM + PC + MSc + IFN – γ group: 10 mice were injected with bone marrow cells and spleen cells, taking 0.5 ml of MSc cells after one hour of IFN – γ tail vein injection; BM + PC group: the remaining 10 mice were not injected with MSC. After treatment, the mice were kept in SPF room.

### Preparation of allogeneic hematopoietic stem cells

Before the experiment, prepare the sterile environment, thoroughly disinfect the experimental equipment, and clean the culture dish. The mice were killed for cervical dislocation, soaked in ethanol, then the spleen was taken aseptically, weighed, filtered and the single cell suspension was collected. The femurs of mice were separated, the marrow cavity was washed with a 1 ml syringe, and the cells were filtered and collected. The erythrocytes were dissolved in 503.1 × g centrifugation, washed twice with PBS, and the ratio of bone marrow cells to spleen cells was adjusted to 1:2. Within 4 hours after TBI treatment, recipients were injected with grafts through tail vein.

### GVHD pathology score

According to the relevant standards, the pathological changes of liver, skin and small intestine in each group were evaluated by GVHD. The pathological score of each mouse ranged from 0 to 10, and 10 was the full score. The score was divided into 3 points of liver change, according to the criteria of liver cell pathology, according to the degree of liver cell necrosis; 3 points of small intestine tissue change, according to the pathological score of small intestine, according to the morphological changes of goblet cells and crypt cells of intestinal wall and the infiltration of inflammatory cells; 4 points of skin change, according to the pathological score of skin, according to the changes of epidermal cells, basal cells and sebum Score of permeability of glands.

### Statistical analysis

SPSS 12.0 software was used for statistical analysis. The data was expressed as mean ± standard deviation (x ± SD). Independent t-test was carried out among samples, and X2 test was carried out for the rate, P < 0.05, which was statistically significant.

## Results

### Condition record of mice after transplantation

After hematopoietic stem cell transplantation, the weight of mice was measured every day, and the diet, hair, mental state, survival, mobility, sensitivity and feces were observed. One week after transplantation, 2 ml of blood was collected from the vein queue every two days to monitor the peripheral blood leukocytes of mice, and the survival time of mice in each group was recorded.

### Weight changes of mice after hematopoietic stem cell transplantation

**Figure 1.**
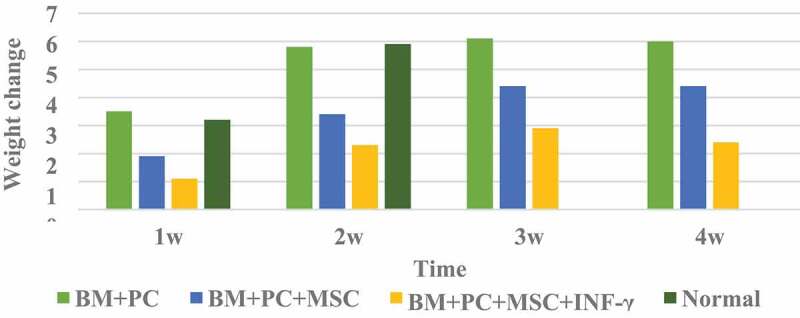
Weight changes of mice after hematopoietic stem cell transplantation

The weight change of mice after hematopoietic stem cell transplantation is shown in Figure 1.

It can be seen from Figure 1 that the body weight of mice in the BM+PC group after HSCT at 2 W, 3 W, and 4 W changed significantly compared with that in the 1 W group; mice in the BM+PC+MSc group and BM+PC+MSc+IFN-γ group after hematopoietic stem cell transplantation There are significant changes in body weight at each stage; Compared with the BM+PC+MSc group without IFN-γ treatment, the weight of the mice in the BM+PC+MSc+IFN-γ group treated with IFN-γ has a greater difference, which proves that IFN -γ has a certain effect on the body weight of mice.

### Effect of mouse MSC on serum IFN-γ level after transplantation

**Figure 2.**
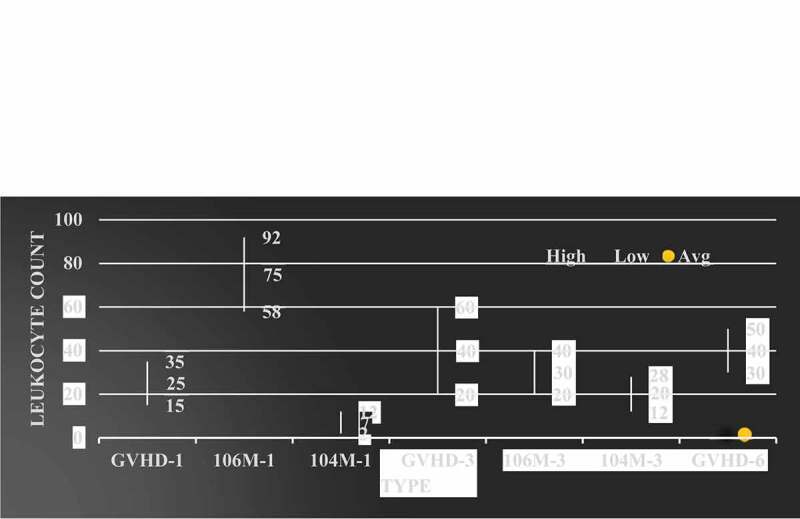
The level of IFN-γ after transplantation

The effect of mouse MSC on serum IFN-γ levels after transplantation is shown in Figure 2.

It can be seen from Figure 2 that compared with healthy mice, the serum IFN – γ concentration in the experimental group is lower, only 2.5 pg/ml, and there is no statistical significance between the serum IFN – γ concentration in the high concentration MSc transplantation group and that in the induction group (P > 0.001); the serum IFN – γ concentration in the low concentration MSc transplantation group has a significant change compared with that in the induction group (P < 0.001); Compared with the induced group, the IFN – γ level in the transplantation group with high concentration of MSCs at 3 W had a smaller change (P < 0.001), which was statistically significant; compared with the normal group, the IFN – γ level in the transplantation group with high concentration of MSCs at 4 W had no significant difference.

### Number of leukocytes in mice after hematopoietic stem cell transplantation

**Figure 3.**
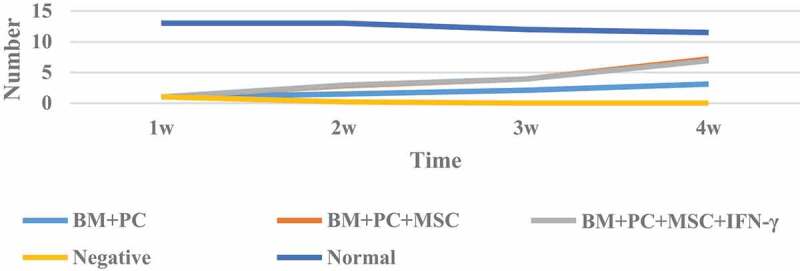
Number of leukocytes in mice after hematopoietic stem cell transplantation

The leukocyte value after mouse allogeneic hematopoietic stem cell transplantation is shown in Figure 3.

It can be seen from Figure 3 that the number of white blood cells in the body of mice after hematopoietic stem cell transplantation has significant changes compared with the normal group at all times; the most is BM + PC + MSc group, and the least is negative group; in the BM + PC group after hematopoietic stem cell transplantation, the total number of white blood cells at 2 W, 3 W and 4 W has great changes compared with the total number of white blood cells at 1 W; There was no significant difference between BM + PC + MSc group and BM + PC + MSc + IFN – γ group in the first week after transplantation, but the number of leukocytes in mice was significantly different from that in BM + PC group in the second, third and fourth weeks after transplantation.

### Comparison of GVHD Clinical manifestations in mice after hematopoietic stem cell transplantation

The clinical manifestations of GVHD after allogeneic hematopoietic stem cell transplantation in mice are shown in Table 1.

**Table 1.** Occurrence, survival and death of GVHD after allogeneic hematopoietic stem cell transplantation in mice

Group	Donor	Recipient	GVHD incidence rate	GVHD mortality	Survival rate	Time
BM+PC	C57BL/6	BALB/c	15/15	15/15	0	28d
BM+PC+MSC	C57BL/6	BALB/c	15/15	6/15	60%	36–40d
BM+PC+MSC+IFN-γ	C57BL/6	BALB/c	18/20	4/18	75%	42–45d
Negative	-	BALB/c	-	-	0	10–13d
Normal	-	-	-	-	100%	-

As can be seen from Table 1, the mice in the control group showed a decrease in food and water intake after radiation. In the first week, mental depression, decreased activity and weight loss occurred. The symptoms were obvious on the fifth day, but after the second week, the symptoms improved and gradually returned to normal. The survival rate within four weeks was 100%. In the allogeneic group, the mice showed symptoms of mental failure and decreased activity. After two weeks of transplantation, the weight of the mice decreased again on the 15th day, and showed different degrees of GVHD, such as decreased activity on the 12th day, diarrhea and vertical hair began to die, and the survival rate on the 24th day was more than 50%. The mice in IFN – γ group showed severe mental depression, back scales and serious hair loss, exposed skin and ulcer, severe diarrhea, bloody stool and even anorectal prolapse. All mice had respiratory distress symptoms before death. The clinical score increased significantly about one week after transplantation, and then continued to rise until death. Four weeks later, four mice died, weighing about 18 g at the time of death, and the rest survived for a long time.

### Pathological score of GVHD after hematopoietic stem cell transplantation

**Figure 4.**
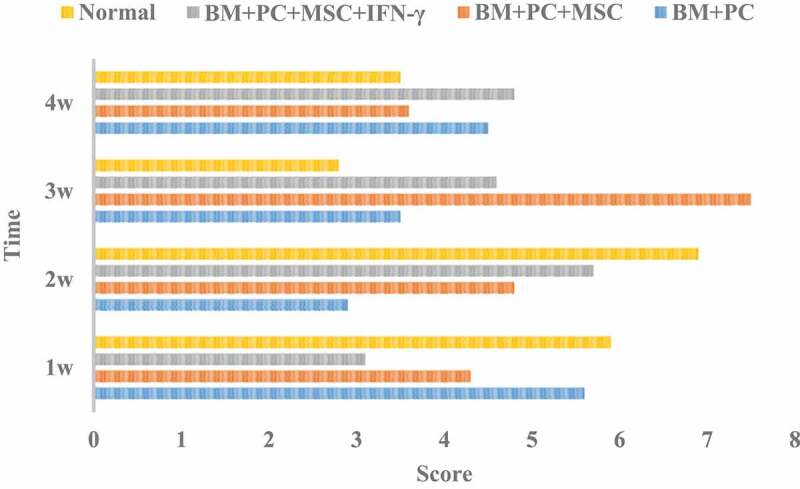
GVHD pathological score after allogeneic hematopoietic stem cell transplantation in mice

The pathological score of GVHD after allogeneic hematopoietic stem cell transplantation is shown in Figure 4.

Analysis of Figure 4 shows that the scores of GVHD after allogeneic hematopoietic stem cell transplantation of mice in each group fluctuated greatly. The score of BM + PC + MSc group was the highest at 3 W, and changed greatly at different times. The average score of BM + PC + MSc + IFN – γ group was the lowest, and the change was also small. After allogeneic hematopoietic stem cell transplantation, the incidence of GVHD in the three groups of mice were tested by x2 test, and X2 = 20.6, which showed that the incidence of GVHD in the three groups of mice changed significantly, and the survival rate increased.

## Discussion

At present, there are many different views on the immunosuppressive effect of mesenchymal stem cells, including two aspects: inhibiting the immune function and promoting the development of immune process. The study of the immune mechanism of mesenchymal stem cells mainly includes the direct interaction between cells and the regulatory factors of solvent. Mesenchymal stem cells can inhibit the proliferation and secretion of T-lymphocytes and B-lymphocytes, which can be used to reduce the incidence of GVHD. However, mesenchymal stem cells do not have or have very low immunosuppressive capacity, and only when stimulated by activated lymphocytes or inflammatory substances secreted by them can they have a strong level of immunoregulation. It is of great significance to understand the relationship between soluble drugs and immune regulation of mesenchymal stem cells.

The use of allogeneic hematopoietic stem cell transplantation is a milestone in the treatment of hematological malignancies, and it is one of the effective means to treat hematological malignancies. The further implementation of this treatment method greatly improves the prediction ability and long-term disease-free survival rate of patients. However, severe graft-related complications, such as host transplant disease (GVHD), are still the main problems limiting its implementation. On the one hand, it seriously affects the quality of life of patients after transplantation, on the other hand, it also reduces the appearance of GVL to a certain extent. Allogeneic hematopoietic stem cell transplantation is a treatment method to break the initial immune balance and restore the new balance. After the establishment of a new immune system, due to the incompatibility of antigens between the donor and the host, the new balance often has a positive immune response, leading to serious immune damage, that is, GVHD. At present, cyclosporine, mycophenolate mofetil and low-dose methotrexate are used to prevent and treat GVHD transplantation. Anti thymocyte globulin, anti-CD25 monoclonal antibody or fludarabine should be given according to the matching degree of HLA and the relationship between donors and recipients. However, these treatment methods are not specific, in the prevention and treatment of GVHD, it will inevitably cause new damage to the human immune system. Therefore, in-depth study of GVHD can provide more ideas and theoretical support for the clinical prevention and treatment of GVHD.

## Conclusion

In this study, GVHD model was successfully established in mice to observe the performance of different recipients after hematopoietic stem cell transplantation. In order to maintain the accuracy of the experiment, we tried to avoid the hematopoiesis stage, so that the donor could be permanently implanted for a long time. It was found that the incidence of GVHD was significantly reduced and the survival rate of mice was significantly increased after allogeneic hematopoietic stem cell transplantation with IFN – γ treated MSCs. This experiment provides a theoretical basis for further experiments, and also provides an experimental basis for clinical transplantation of allogeneic hematopoietic stem cells.
